# Caregiver perceptions of the broader societal benefits of vaccination: A path toward sustainable vaccine advocacy in India

**DOI:** 10.1016/j.ssmqr.2022.100156

**Published:** 2022-12

**Authors:** Baldeep K. Dhaliwal, Ananya Rattani, Riti Chandrashekhar, David E. Bloom, Anita Shet, Rajeev Seth

**Affiliations:** a Johns Hopkins Bloomberg School of Public Health, Department of International Health, 615 N. Wolfe Street, Baltimore, MD, USA; b Bal Umang Drishya Sanstha, E-10, Green Park Main, New Delhi, India; c Harvard T.H. Chan School of Public Health, 665 Huntington Avenue, Boston, MA, USA

**Keywords:** Qualitative, Vaccines, Public health, Immunization, Vaccine advocacy

## Abstract

Over the last decade growing public health evidence suggests that, in addition to health-related benefits, there are also social and economic benefits of vaccination. Research to understand how caregivers in low-and-middle-income countries perceive these social and economic benefits, or if these benefits factor into their vaccination decisions for their children, has been limited. Leveraging qualitative strategies to gain more nuanced insights into caregiver perceptions of vaccination benefits has also been significantly underexplored. We conducted in-depth interviews with 13 caregivers of children, at which point we reached saturation, in Mewat District, Haryana, an area in India with low vaccination coverage. Interview results suggest that caregivers of children associate positive health outcomes with vaccination programs, and some additional social and economic benefits beyond improved health outcomes. Caregivers also shared how local advocacy and gaps in vaccination programs can affect their perceptions of vaccination benefits. Qualitatively exploring the perceived benefits provides a unique understanding of the value that caregivers assign to vaccination and complements existing knowledge on factors that dissuade caregivers from vaccination. These insights will allow researchers to better identify and design context-specific advocacy strategies to strengthen vaccination programs in communities with low vaccine uptake and acceptance.

## Highlights

• Growing literature suggests vaccination services provide social and economic benefits in addition to disease prevention.
• Research exploring if caregivers perceive vaccination benefits, or if these affect vaccination decisions, has been limited.
• Findings suggest caregivers associate positive outcomes, and some social and economic benefits, with vaccination programs.
• These data add a unique perspective, as they qualitatively explore factors encouraging caregivers to vaccinate children.
• This underexplored domain complements studies exploring factors that dissuade caregivers from vaccinating their children.

## 1 Background

Vaccination programs are one of the greatest health achievements, averting an estimated 2–3 million deaths globally each year and effectively preventing infectious diseases (Andre et al., 2008; World Health Organization, 2019). India in particular has made tremendous progress toward improving vaccination coverage across the nation's annual birth cohort of 27 million (Bettampadi et al., 2021). India's Universal Immunization Program, launched in 1985, has continued to grow stronger by adding newer vaccines, improving self-sufficiency in vaccine production, improving cold-chain and logistics management, and strengthening surveillance for vaccine-preventable diseases (Murhekar & Kumar, 2021). India also introduced national programs such as Mission Indradhanush in 2014 to achieve high levels of immunization coverage, enhancing this to Intensified Mission Indradhanush in 2018. From initial vaccination tracking in National Family Health Survey-1 (1992–1993), to the most recent vaccination tracking in National Family Health Survey-5 (2019–2021), the percentage of fully vaccinated children across India has increased from 35.5% to 76.4%.

Despite this progress, the goal of equitably vaccinating the nation's children remains elusive,3 as the country still accounts for a third of global under-five deaths (United Nations Inter-agency Group for Child Mortality Estimation, 2020), with many of these deaths occurring in India's most under-immunized areas. In Mewat District in Haryana State (Image 1), only 53.8% of children under two years of age were reported to be fully immunized with bacilli Calmette-Guerin, measles, and three doses each of the polio and diphtheria-pertussis-tetanus vaccines based on India's National Family Health Survey-5 conducted from 2019 to 2021 (National Family Health Survey-5, 2021). In addition to understanding factors associated with vaccination uptake, such as acceptance, hesitancy, and misinformation, understanding perceptions of health-related benefits associated with vaccines may provide more nuanced insights on vaccination rates. Understanding these perceptions in areas such as Mewat may provide insight into identifying gaps and effective strategies to improve vaccine uptake in areas with low vaccine coverage across India.

In addition to disease prevention, a growing body of evidence suggests significant social and economic benefits are also associated with vaccination programs. Vaccination programs have been associated with reduced healthcare costs, improved educational attainment and cognitive performance, and improved equity in health and financial benefits across social strata, among other benefits (Bloom et al., 2011; Deogaonkar et al., 2012; Verguet et al., 2013).

Researchers have largely employed quantitative methods to understand how caregivers perceive these social and economic benefits of vaccination. The use of qualitative methods to understand health-related, and social and economic, benefits of vaccination programs from a caregiver perspective has been more limited. Traditionally, qualitative methods have been leveraged for understanding other perceptions of vaccination, such as factors associated with acceptance, misinformation, barriers, and hesitancy. While understanding these more traditional behavioral factors through a qualitative lens is critical, qualitatively understanding perceived benefits of vaccination and vaccination programs is underexplored in low-and-middle-income countries. Investigating the experiences and perspectives of caregivers qualitatively, particularly in settings with low vaccine uptake, provides a unique understanding of specific social values that caregivers associate with vaccination programs. Qualitatively exploring perceptions of benefits is particularly timely as current disruptions to health services due to the COVID-19 pandemic threaten to further deepen existing inequities and gaps in vaccination services (Murhekar & Kumar, 2021; United Nations Inter-agency Group for Child Mortality Estimation, 2020). While the selected geographic area of study, Mewat, has lower vaccination rates than the national average in India, the expectation is that these caregivers will provide insights into any benefits that caregivers most associate with vaccination that are transferrable across other settings with low vaccine uptake.

## 2 Methodology

### 2.1 Study procedures

We conducted in-depth interviews between September and November 2020.

Researchers at the [details omitted for double-blind reviewing] and [details omitted for double-blind reviewing] developed a semi-structured in-depth interview guide to assess caregivers’ perceptions of the social and economic benefits of vaccination (Appendix 1). Our overall domains encompassed caregiver personal experiences with vaccination, caregiver clinic experiences, and perceived health and broader benefits of vaccination.

We drew participants from a larger quantitative pilot study in Mewat focusing on performance of children on cognitive tests, their vaccination history, and other related parameters. This quantitative pilot study aimed to establish the feasibility of a longitudinal study assessing these parameters. Records from Anganwadi centers, or rural childcare centers, were used to create lists of children aged 18 months to 8 years in the Ghasera village of Mewat for the quantitative study. Anganwadi records were selected, as they are traditionally used to make vaccination lists for healthcare workers.

During the recruitment process for the in-depth interviews, research assistants explained that interviews would be conducted with whomever in the household was considered the “primary caregiver” of the child. For the purpose of this paper, the definition of “caregiver” encompasses mothers, fathers, and grandparents of children enrolled in the larger study, who were considered primary caregivers of the enrolled child.

We trained local research assistants to facilitate in-depth interviews. These research assistants were selected as they were well-known in the community, they have previously facilitated similar qualitative research in Mewat, and they have been previously involved with weekly mobile health clinics for residents. They were provided additional training on qualitative techniques, including when to identify when data saturation had been reached.

We made reminder calls and sent texts prior to the interviews, and the interviews were conducted at one Anganwadi Center in Ghasera, Mewat. Trained research assistants led the in-depth interviews in Hindi, along with a local [details omitted for double-blind reviewing] staff member who could facilitate specific translations in the local dialect if required.

### 2.2 Participants

Participants were selected through purposive sampling, based on the criteria of being eligible for the larger quantitative study, as well as their experiences with vaccination services. In-depth interviews were recorded digitally. Prior to conducting in-depth interviews, participants provided written informed consent in Hindi. If a participant could not read, a research assistant read the consent form out loud to them in Hindi and answered any questions. Similarly, if a participant could not write their name, a thumbprint was taken while a witness observed to ensure all participant questions were answered. After the participant's thumb impression was taken, the witness also added their signature to the consent form. All identifiers and information involving personal data were removed from the transcribed data to keep the identities of the participants anonymous, and to maintain confidentiality.

Thirteen in-depth interviews were conducted. At this point, research assistants noted that they had been hearing similar responses from participants and new data were not emerging. When research assistants felt confident that saturation had been reached, they stopped data collection. Participants included 11 mothers, 1 father and 1 grandmother of children.

### 2.3 Analyses

The recordings were in Hindi and Mewati dialects, which were transcribed and translated into English by an external translation company. Initial transcripts were reviewed by the research assistants who conducted the interviews to ensure words and meanings were accurately captured in English. To further ensure the translations were accurate, research assistants listened to audio recordings and ensured colloquialisms in Mewati, were accurately captured. Three individuals developed codes for the transcripts. Two individuals were based in India and were involved in primary data collection, and one individual was based in the United States and was not involved in primary data collection. All individuals involved in data analysis have previously collaborated on qualitative data analysis and have backgrounds in public health. The team allocated codes and subcodes to the transcripts using a grounded theory approach (Noble & Mitchell, 2016). The team reviewed codes, established consensus on data interpretation, and discussed emergent themes on a weekly basis to build consensus. Lastly, the team organized quotes to demonstrate the emergence of themes across multiple transcripts.

### 2.4 Ethical considerations

Participant recruitment began after obtaining approval from [details omitted for double-blind reviewing] [details omitted for double-blind reviewing]. We obtained ethical approval from Health Ministry Screening Committee (2020–4628/F1**).** Separate ethical approval was obtained from the [details omitted for double-blind reviewing] to ensure context-specific COVID-19 safety protocols were designed for safe data collection.

## 3 Results

The themes that emerged in our results support four main messages. First, caregivers of children associated several positive health outcomes with vaccination programs. Second, caregivers felt that vaccination programs provided them with benefits beyond access to vaccines. Third, caregivers credit local advocacy and familial support as playing a role in their ability to decide to utilize vaccination programs for children. Last, caregivers perceive gaps in vaccination programs, which negatively impacts their perceptions of vaccines and healthcare workers.

### 3.1 Health-related benefits of vaccination programs

Most participants believed that vaccination programs prevent their children from contracting specific diseases, including polio and tuberculosis. Although participants’ specific knowledge of the diseases that vaccines protect against differed, participants broadly understood that vaccines prevent severe childhood disease. Vaccination programs in the community were also credited with improved community knowledge of diseases and the specific diseases that vaccines prevent.

*“In the future, if there is TB [tuberculosis] but there is [vaccination], then they are [protected] when they are young. Then there is nothing to worry about [regarding TB] in the future.”*

In addition to disease reduction, participants cited broader health-related benefits and knowledge that they also associated with vaccination programs and vaccinators.

*“They give medicines when they vaccinate on the shoulder or the thigh. [They say]it’s for reducing fever.”*

Although vaccination programs are primarily targeted toward children, the services also provide vaccines and antenatal care to pregnant women. Participants shared that this type of advice and guidance would not be easily accessible to residents of Mewat without childhood vaccination clinics.

*“[When we are there] they ask us if there’s any issue that we are facing. If pregnant then [they] ask about the [baby’s] movements, and she explains everything well.”*

#### 3.1.1 Social and economic benefits of vaccination programs

Participants highlighted the social and economic benefits that they also associated with vaccination programs. They expressed that they receive nutritional supplements, door-step general informational services, and educational benefits for themselves and their children.

Participants shared that when they take their children to vaccination clinics, they also receive food rations. On days when vaccines are not provided, children can still get food for their morning meals.

*“Benefits [of the vaccine programs] are they get vaccines, but [Community Health Worker] gives our children utensils, food, and [she] call[s] them here to study.”*

Caregivers shared that when vaccinators come door-to- door they provide knowledge to caregivers, not only about the vaccination program, but also about general guidance on maintaining health.

*“She asks us to move around only in the house, so the child and mother both are healthy. When a child is born, she explains everything [to stay healthy].”*

Participants also noted that their children receive education services at these vaccination centers through teaching sessions. During these teaching sessions, children learn counting and reading skills, and receive nutrition information. They also develop essential social interaction skills with other children while they are at these teaching sessions.

*“Children are looked after, [and] they study and play [at the Anganwadi center].”*

Implementing vaccination programs at the Anganwadi center where educational services are provided increases opportunities for children to be exposed to educational programs and allows children in educational programs to access vaccination programs.

#### 3.1.2 Role of local advocates, religious leaders, and families in vaccination

Caregivers shared that local community-focused outreach assists them with understanding the purpose of vaccination programs, addressing fears, and overcoming logistical access barriers. Participants specifically stated that when their local community health worker advocates for vaccines, they are more likely to accept them.

*“Whenever they come for vaccination, we ask Sapna [Anganwadi worker] about it. We only go for it after asking her.”*

Participants also explained that when influential members of their community, such as their father-in-law, religious leader, or mother-in-law, advocate for vaccination, they are more likely to take their child for vaccination.

*“My father-in-law is a doctor, so he vaccinated my oldest child. He shared that it’s good to get it done.”*

Local community advocacy was also credited as being influential in vaccination decisions among caregivers. When other members of the community, such as neighbors and teachers, accept and encourage vaccination, others become more likely to take their children to receive vaccines.

*“My neighbors had children before me. They told me that the vaccines were good and told me to get my children vaccinated.”*

#### 3.1.3 Gaps in vaccination programs

Although caregivers shared the benefits that they associate with vaccination programs, they also noted there are existing gaps which negatively impact their perceptions of programs. Participants noted several logistical challenges associated with vaccination programs. When services were far away, women found it too difficult to access vaccines.

*“My eldest daughter didn’t receive [vaccines] because the Anganwadi was far away, and [the closer one] didn’t provide vaccination at that time.”*

Additionally, many women shared that they travel back to their maternal homes shortly after childbirth, contributing to reduced adherence to the vaccination schedule and an increase in missed vaccination early in the child's life. When women who have given birth travel home to their maternal homes, they may forget to bring their child's vaccine card, they may not know where vaccination clinics are being held, and their older children, who stay at their married homes, may also miss vaccination appointments while mothers are recovering from childbirth in their maternal homes.

Some participants also shared that they feel vaccinators do not provide detailed information on the benefits of vaccination, instead expecting caregivers to simply vaccinate their child.

*“I was angry because she said it’s for the children’s benefit, but she never clarified the benefits.”*

Last, female participants felt that the responsibility of getting their child vaccinated, and caring for the child after they were vaccinated, rested with them. Men were noted as having less of a role in accessing vaccines for their children.

*“My wife goes [to the vaccination center] … because I’m usually busy, and I cannot look after a [sick] infant.”*

## 4 Discussion

Previous research indicates that vaccination benefits go beyond disease prevention, with specific vaccines even offering cross-protection against diseases not specifically targeted by the vaccine itself (Franklin et al., 2020). Our qualitative data suggest that caregivers of children also perceive some additional health, and some social and economic benefits, providing an additional qualitative perspective to the literature highlighting social and economic benefits (Dhaliwal et al., 2021; Nandi, Shet, et al., 2019, Nandi, Deolalikar, et al., 2019; Canning et al., 2011). Further, our data also point to the importance of designing effective communication strategies about vaccination programs, identifying local advocates, and using context-specific language to communicate benefits to caregivers, complementing existing data (Dutta et al., 2020, 2021).

Context-specific advocacy strategies could be leveraged to bring further social and economic benefits of vaccination programs to the attention of caregivers. Previous research has indicated that in 39–91% of childhood rotavirus gastroenteritis cases, at least one parent was required to take time off work (Van der Wielen et al., 2010). Although, many caregivers interviewed discussed the negative impact of side effects from vaccines have on their day-to-day routine and daily wages, the loss of productivity and income associated with caring for a child who is sick from a vaccine-preventable disease could be substantially higher for caregivers. As many residents in rural communities, such as Mewat, are daily wage earners, training local advocates to discuss long-term economic costs associated with missed vaccination may motivate uptake. Designing communication strategies with local champions may produce long-term advocates within the community who understand the importance of vaccination programs, and the full benefits of vaccines may better leverage effective strategies in their advocacy.

These advocacy and communication strategies are particularly critical considering the sharp decline in routine immunization services in India during the COVID-19 pandemic (Shet et al., 2021). Research in India has shown that 83% of surveyed providers reported substantial decline in vaccination services in April–June 2020, with only 38.7% of surveyed providers having a plan to initiate catch up services. Even as modest declines in vaccination rates compromise community immunity, outbreaks of vaccine-preventable diseases in pockets across the globe continue to emerge (Bahl et al., 2021). Context-specific strategies designed to improve routine immunization may also be effective in strengthening the COVID-19 vaccination program among adults and, eventually, among children in India.

India has successfully vaccinated 74% of its 18+ population with their first dose of the COVID-19 vaccine, roughly 67% of the 18+ population with two doses, and only 3.3% of the 18+ population with an additional (booster) dose (Holder, 2021). In light of this decline between first and second doses, limited booster doses, and the upcoming approval for COVID-19 vaccines for children, using grassroots-level strategies, leveraging local knowledge and terms, and harnessing the strength of local advocates, as identified by our research, may be essential to expanding COVID-19 vaccine uptake in rural communities.

Several factors limit this study. Our sample of caregivers represents a subset of caregivers from a larger study, whereas recruitment from villages across Mewat might have allowed us to have a wider set of participants. These participants were also from the same village and were predominantly women, limiting the type of perspectives we received. Had we recruited from multiple villages and included more males, we may have had more diverse perspectives. We also recognize that our definition of “caregiver” may be limited, as other household channels of influence may exist, including aunts and uncles in joint households. Selection bias may have influenced the type of responses constituting our findings, as the participants may be strong advocates for vaccination programs themselves, such that opposing views may not have been elicited. Another limitation with our in-depth interviews was that we conducted them during the first wave of COVID-19 cases in India. Caregivers may have felt uncomfortable with interviewers due to social distancing and personal protective equipment requirements, that prevented them from speaking freely with interviewers.

Nevertheless, our qualitative findings underscore some social and economic benefits of vaccination services perceived by end-users of vaccines, and illuminate a path for long-term vaccine advocacy. Our findings also further emphasize the need for additional research that leverages qualitative methods to explore perceived benefits of vaccination programs from the caregiver perspective. While qualitative studies exploring hesitancy and factors dissuading caregivers from vaccinating their children are abundant, studies exploring the benefits that caregivers associate with vaccination programs have been limited.

## 5 Conclusion

Traditionally, understanding caregiver perceptions of health-related and social and economic benefits of vaccination has been from a quantitative perspective. Leveraging a more nuanced qualitative lens, as in this study, provides a more well-rounded perspective of caregiver perceptions of vaccination programs, particularly the benefits of vaccination services. Utilizing this perspective enables us to identify specific, bottom-up strategies that leverage unique insights to facilitate long-term improvements in routine and COVID-19 vaccine uptake and acceptance. Gaining this rich detailed knowledge can provide tools to help remove entrenched obstacles to vaccination acceptance, address vaccine equity, and strengthen vaccination programs in communities where most children remain vulnerable to childhood diseases.

## Authors’ contributions

BD and AS conceptualized and designed the study. BD, RC, and AR analyzed and interpreted the data and were major contributors to the manuscript writing. RC and AR facilitated data collection. DB, AS, and RS were critical contributors to writing the manuscript. All authors read and approved the final manuscript.

## Ethics approval and consent to participate

We obtained ethical approval for the study from the Johns Hopkins Bloomberg School of Public Health Institutional Review Board and the Ethics Committee of Bal Umang Drishya Sanstha. Written consent was collected from all participants prior to engaging in data collection.

## Funding

This work was supported by the Value of Vaccination Research Network through a grant from the 10.13039/100000865Bill & Melinda Gates Foundation (Grant OPP1158136). The content is solely the responsibility of the authors and does not necessarily reflect the views of the Value of Vaccination Research Network or the foundation. The funding bodies played no role in the design of the study; in collection, analysis, and interpretation of data; and in writing the manuscript.

## Declaration of competing interest

The authors declare that they have no known competing financial interests or personal relationships that could have appeared to influence the work reported in this paper.
