# Supplementary material for: Caregiver perceptions of the broader societal benefits of vaccination: A path toward sustainable vaccine advocacy in India
Source: SSM Qual Res Health. 2022 Dec;2:None. doi: 10.1016/j.ssmqr.2022.100156 (PMC9748304; doi:10.1016/j.ssmqr.2022.100156)
Supplement: Multimedia component 1 [file mmc1.docx]

**Appendix 1: In-Depth Interview Guide**

**Background for Interviewer**

The questions are designed to allow the participant to describe what their experiences with vaccinations have been like in the clinic setting, from their own personal experiences, and in the context of the community. These questions (in **BOLD**) are designed to maintain the conversation. After each question, in brackets, is a list of probes that may or may not be used to prompt caregivers to continue their conversation. You do not need to use every probe in the guide; the probes are there to remind you to consider those dimensions when listening to the caregiver. We need to hear from the caregiver as much as possible. It is important not to suggest answers or ask questions in a way that leads the caregiver to a particular answer.

Please take notes during the interview and summarize your general impressions at the end of the interview.

**Please read text below to caregiver**

The purpose of this interview is to learn about your experiences and your thoughts on vaccinations that your child may or may not have received. We would like to hear about your experiences that drove your decisions during the immunization process, experiences with vaccinations, and what benefits and risks you associate with vaccinations. As we discussed during the consent process, I will be taking notes on what you say, and I would like to record the interview so that I can make sure that I don’t miss anything you say. Your name will not be recorded. The recording will not be shared with anyone outside of the study team and will be destroyed after the study is complete.

**Domain 1: Caregiver Personal Experiences**

1. What do you think are the benefits of vaccinations, or the benefits of the vaccination clinics?
   1. Follow up: If none, why do you think so?
   2. Follow up: Do you think are problems with vaccines, or the vaccine clinics?
2. Are there benefits of vaccines other than avoidance of disease?
   1. Probe: child care, sick child care, child feeding practices, hygiene (such as washing hands), child safety, and education for your child, nutritional supplements, and social interaction benefits
3. How did the information provided by health personnel during your visits to vaccination clinics impact on your decision about giving vaccines to your child?
4. What experiences motivate you to provide vaccinations to your child?
   1. Probe: How was it the first time you got your child vaccinated; who motivated you; were there specific experiences that motivated you to get your child vaccinated?
5. What is your view on how important vaccinations are for other children?
   1. Probe: Does another child’s immunization decision impact you or your child; Do you feel immunizing your child will impact other children?

**Domain 2: Caregiver Clinic Experiences**

1. Can you tell me about your experiences while obtaining vaccinations?
   1. Probe: interaction with provider; waiting time, access, you child vaccination status, place of vaccination, frequency
2. How were the attitudes of the healthcare providers at the vaccination clinic?
   1. Probe: Respectful? Do they explain things nicely?
   2. Do you get to ask questions that are in your mind? And are they answered fully?
3. What kind of information do you get from the immunization clinics, which you would **NOT** be able to access otherwise?
4. What experiences do you **not like** about coming to the vaccination clinics?
5. Have you observed any child get harmed or have any bad experiences with vaccinations? If yes, please explain and how did you come to know about this?
   1. Probe: discussions, word of mouth, clinic warnings?
