# Supplementary figures and images for: Caregiver perceptions of the broader societal benefits of vaccination: A path toward sustainable vaccine advocacy in India
Source: SSM Qual Res Health. 2022 Dec;2:None. doi: 10.1016/j.ssmqr.2022.100156 (PMC9748304; doi:10.1016/j.ssmqr.2022.100156)

Image 1: Map of Mewat, Haryana


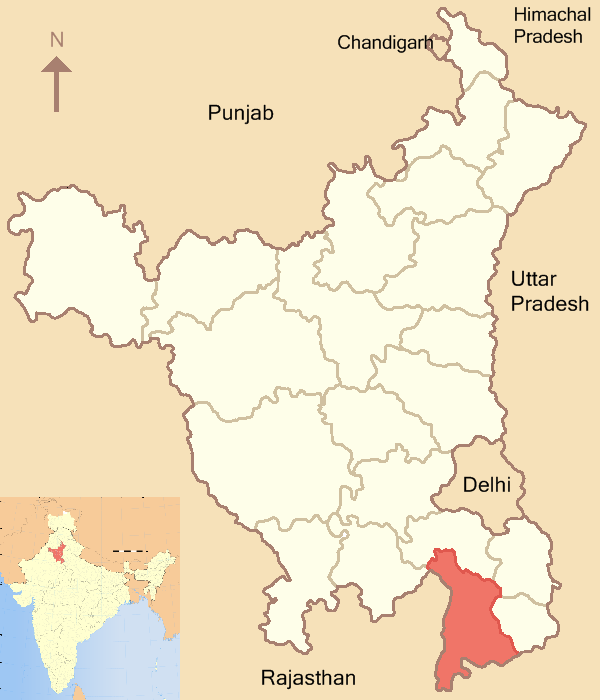

Supplement: Multimedia component 2 [file mmc2.docx]
